# Quasi-Randomized Trial of Contact With Nature and Effects on Attention in Children

**DOI:** 10.3389/fpsyg.2019.02652

**Published:** 2019-12-05

**Authors:** Shannon A. Johnson, Stephanie Snow, Michael A. Lawrence, Daniel G. C. Rainham

**Affiliations:** ^1^Psychology and Neuroscience, Dalhousie University, Halifax, NS, Canada; ^2^School of Health and Human Performance, Dalhousie University, Halifax, NS, Canada

**Keywords:** nature contact, children, attention, restoration, urban

## Abstract

Children today spend less time in nature than previous generations and there is concern that this shift negatively impacts children’s cognitive abilities, particularly their ability to direct their attention. Theories, such as the Attention Restoration Theory (ART), suggest that contact with nature may replenish endogenous attention (e.g., directed, voluntary attention). There is a lack of rigorous research on how contact with nature is associated with attentional performance in children. This study employed a quasi-experimental design and included a sample of typically developing children to investigate performance on computerized endogenous and exogenous attention tasks before and after exposure to one of two interventions – a 30-min walk in either an urban (*n* = 30) or natural (forested, *n* = 30) environment. The two experimental groups were equivalent with regard to sex ratio, age, IQ, and connectedness to nature. Attention was assessed using the Combined Attention Systems Test (CAST), a state-of-the-art assessment tool designed to evaluate exogenous and endogenous attention characteristics. Bayesian hierarchical modeling of both response time (RT) and error rate (ER) was employed to evaluate the fixed effect of attentional measures and interactions with session and group. Consistent with predictions of ART, results support credible effects of the nature intervention on two measures of endogenous attention: Alerting RT: *d* = 0.85 (95% CI: 0.21–1.8), Orienting ER: *d* = 1.45 (95% CI: 0.17–7.18), but not on any of the measures of exogenous attention.

**Clinical Trial Registration:** https://www.isrctn.com/, identifier ISRCTN17762011.

## Introduction

Modern lifestyles of children are predominately urban and indoors, disengaged from outdoor experiences with natural environments, and in contrast with psycho-evolutionary theories espousing contact with nature as crucial for healthy physical and cognitive development. Eighty-two percent of North Americans currently reside in urban settings (United Nations, 2014). Thus, even when children spend time outdoors, it is unlikely to be a “natural” environment. More than ever before, people are insulated from regular contact with nature (Maller and Townsend, 2006; The David Suzuki Foundation, 2012).

This modern separation from nature is problematic because many studies with adult samples have indicated that exposure to nature is associated with improved well-being (Ulrich et al., 1991; Kuo and Faber Taylor, 2004; Staats and Hartig, 2004; Pretty et al., 2005), more positive perceptions of quality of life (Ogunseitan, 2005), and improved self-esteem (Staats and Hartig, 2004). The health-enhancing properties of nature may be mediated through several mechanisms, including recovery from stress (Kuo and Faber Taylor, 2004), facilitation of social contact (Leyden, 2003), physical activity (Pretty et al., 2005), and the restoration of attentional resources (Kaplan and Kaplan, 1989; Hartig et al., 1991). Although research clearly supports positive influences of nature exposure in adults, there has been less research examining how children are affected by exposure to nature. Louv (Louv, 2005) coined the term “nature deficit disorder” to capture the potential cognitive, psychological, and physical problems associated with disconnection from nature in children. Despite growing evidence that nature enhances health and awareness of the potential problems associated with disconnection from nature, there has been little systematic study of how time spent in nature affects children.

One of the major theories regarding the mechanisms through which nature benefits human well-being is the Attention Restoration Theory (ART). This theory is predicated on the notion that attention, particularly directed (i.e., effortful, voluntary) attention, is a finite resource and susceptible to fatigue under conditions of prolonged use (Olmsted, 1968; Kaplan, 1995; Wells, 2000; Kaplan and Berman, 2010). That is, attentional capacities become fatigued due to the many demands placed upon them in our everyday life. This fatigue manifests itself as an inability to concentrate and ignore distracting stimuli, and may lead to irritability and anxiety particularly in children. Time spent in restorative environments can render the need for directed attention temporarily unnecessary and, thus, allow attention to rest and replenish (Kaplan, 1983). Particularly restorative environments are those that engage *soft fascination*. That is, the “attention grabbing stimuli” in these environments must draw gently on our involuntary attention, so as not to overwhelm the attentional system. Thus, nature (e.g., natural parks, forest, the wilderness), which is inherently rich in stimuli that engage *soft fascination* (e.g., ripples on the surface of a lake, rustling leaves, butterflies, song birds), might be particularly restorative (Kaplan, 1995).

Understanding if and how attention in children can be restored is critical, particularly in the context of learning and school performance, as well as associated outcomes including self-esteem, mood, and behavior. Traditional educational approaches provide little time outside of the classroom and minimal opportunity for exposure to nature. The demands on directed attention are high and it is likely that many children experience attentional fatigue during a typical school day. If time outdoors, and specifically time in nature, provides some restorative benefits, this may offer opportunities for educators, parents, and professionals who work with a wide range of children to maximize their learning and other outcomes. Moreover, understanding the links between nature exposure and attention in children is a starting point for uncovering the mechanisms by which exposure to nature positively affects humans.

Over a century ago, William James proposed that there are two types of attention: involuntary attention and voluntary attention (James, 1892). *Involuntary attention* is automatic, effortless, autonomous, and stimulus driven (Kaplan, 1983; Klein and Lawrence, 2012). It occurs when intriguing stimuli reflexively capture our attention (e.g., when we orient to a bright light in the dark) because at some point in evolution it was adaptive to do so, or because of highly specialized training (Kaplan, 1995). James provided a list of the kinds of stimuli that may involuntarily capture our attention: “strange things, moving things, wild animals, bright things, pretty things, metallic things, words, blows, blood” (p. 88). In contrast, *voluntary attention* is non-reflexive. It is primarily an inhibitory mechanism that requires effortful processing and voluntary cognitive control. It is goal-oriented and contingency-based, occurring when we must focus on tasks that are not, in and of themselves, inherently attention grabbing (e.g., attending to instructions). Henceforth, we will use current terminology, endogenous and exogenous, which are based on current theory (Klein and Lawrence, 2012). Conceptually, these terms are largely consistent with, although not synonymous, voluntary and involuntary, respectively.

Theorists suggest that our relative reliance on endogenous and exogenous modes of attention has evolved over time (Kaplan and Kaplan, 1989). For much of human history, exogenous modes of attention served an important survival function. For example, it was likely adaptive to be drawn to attention-grabbing stimuli (e.g., moving things, wild animals, bright things) for daily living skills such as hunting and personal safety. In the context of urbanization, the demands on endogenous attention have increased for individuals across all age groups, and consequently the adaptiveness of, and our reliance on, exogenous attention has decreased (Kaplan and Berman, 2010). In fact, exogenous attention can often create difficulties for us. We are often rewarded for inhibiting exogenous attention (i.e., ignoring the inherently interesting) in lieu of accomplishing our goals (i.e., the modern day “important”) and staying “on task.” The ability to engage in endogenous attention has become increasingly important and adaptive (Bratman et al., 2012). From classrooms to boardrooms, the demands on our endogenous attention are increasingly prolonged, and success in today’s world is often predicated on our ability to meet these demands. However, endogenous attention is a finite resource and susceptible to depletion. The established role of endogenous attention in many facets of daily functioning underscores the importance of identifying ways to remediate and replenish this critical cognitive ability in the face of inevitable depletion.

Attention is also linked to the notion of soft and hard fascination. *Soft fascination* has been contrasted with *hard fascination*, which involves stimuli that abruptly and harshly grab involuntary attention (Kaplan and Kaplan, 1989; Berman et al., 2008). For example, modern urban environments are thought to be non-restorative, as they are characterized by stimuli that evoke *hard fascination* (e.g., car horns, stoplights). Navigating urban environments frequently requires one to draw on rules and contingencies, the implementation of which frequently evokes directed attention. Previous research corroborates, at least in adult populations, the notion that nature might be more restorative than urban environments (Herzog et al., 1997; Hartig and Staats, 2006; Gladwell et al., 2012). For example, in studies designed to probe the restorative features of nature, participants rated images of natural environments as more restorative than images of urban environments (Gladwell et al., 2012), and attentionally fatigued participants were more likely to opt for a walk in a forest than a walk through a city center following a mid-afternoon university lecture (Hartig and Staats, 2006).

Research also suggests that time spent in or viewing images of natural and urban environments differentially affects physiological indices. For example, compared to urban scenes, natural scenes more rapidly returned heart rate (Laumann et al., 2003) and blood pressure (Chang et al., 2008) to baseline following stress induction. Also, walks through natural environments, but not walks through built environments, have been shown to decrease noradrenaline levels (Tennessen and Cimprich, 1995), and vagal activity has been shown to increase when viewing images of natural environments (Herzog et al., 1997), effects suggested to index increased relaxation.

The introduction of ART has fostered a growing body of multidisciplinary research seeking to examine the relationship between time spent interacting with natural environments and attention (Kaplan, 1995; Tennessen and Cimprich, 1995) using various methods and populations (i.e., clinical and typical). In a study of the restorative effect of a wilderness vacation on participants’ abilities to complete a proofreading task (i.e., an indirect measure of attention), participants were randomly assigned to spend time in a natural environment, urban environment, or to engage in passive relaxation (Hartig et al., 1991). Only individuals who went wilderness backpacking showed improvement on second administration of the proofreading task (post-exposure) compared to the other two conditions.

More recent work using more sophisticated methods has corroborated previous findings using a set of well-designed within-subjects quasi-experimental studies that tested ART in a sample of college students (Berman et al., 2008), and a sample of individuals (mean age = 26 years) with Major Depressive Disorder (Berman et al., 2012). In the first study, college students showed significantly greater improvement on working memory tasks after walking in the natural environment compared to walking in the urban environment. Participants in the second study exhibited significant improvements in memory span after a nature walk relative to a walk in an urban environment. Similar studies of adult samples have subsequently found that even minimal exposure to nature (e.g., a view from a window; or plant-presence in a room) and virtual/simulated nature (Ulrich et al., 1991; Tennessen and Cimprich, 1995) can be restorative (Lohr and Pearson-Mims, 2000). However, outcome measures used in these studies do not map on to current conceptualizations of endogenous attention, but instead likely tap several complex cognitive processes including, but not limited to, endogenous attention.

A study to examine the effect of exposure to projected slides of images of urban or natural environments on attention incorporated a between-subjects, pre-post design (Berto, 2005). Participants (mean age = 23 years) first completed the sustained attention to response test (Manly et al., 1999), a 5-min response control test. Next, they viewed 25 images of urban or natural environments, which were each presented for 15 s, before completing the attention task for a second time. The results indicated that after viewing the images of natural environments, participants responded significantly faster, were better able to detect the target, and made significantly more correct inhibitory responses. These same improvements were not observed for participants assigned to view urban images. However, at post-image viewing, participants in the urban group did display a significant reduction in the number of incorrect responses.

Previous research has also examined the implications that time spent interacting with nature, or simply in the presence of nature, has on children’s attention. Several studies have examined the relationship between school-based nature exposure and school performance. For example, it was found that the amount of nature visible through a school’s cafeteria windows, and the objective measure of the amount of vegetation on campus, significantly predicted better performance on standardized testing, higher graduation rates, and higher rate of plans to attend college, even after controlling for socio-economic status, ethnicity, building age, and the size of a given school (Matsuoka, 2010). Another study employed a quasi-experimental design to examine how children’s mood and school behaviors (number of hours of sick leave, record of misbehavior, and academic performance) were affected when classrooms were provided with six medium-sized plants (versus classrooms with no plants) (Han, 2009). The presence of plants in the back of a classroom positively impacted student affect, reduced their number of hours of sick leave, and improved classroom behavior.

Researchers have also sought to examine the relationship between near-home nature and attention in children. A study employing a longitudinal pre-move/post-move design examined the relationship between the naturalness of children’s (age range 7–12) homes and parents’ ratings of their children’s attention (Wells, 2000). Children whose homes improved the most pre-move to post-move on the objective rating of naturalness were rated as having the best levels of attentional functioning. Similar research examined the relationship between parent’s ratings of the views of nature from home and objective measures of children’s cognitive performance (Taylor et al., 2002). For females, parent-rated near-home nature accounted for 20% of the variance in children’s cognitive performance across tasks. Interestingly, no such effect was observed in males. The authors hypothesized that boys do not spend a significant amount of time in the environments around their house.

Consistent with findings indicating relationships between nature exposure and better attention in adults, the majority of child studies drawing on ART highlight the potential benefit of time spent in nature on children’s attention. However, **t**he existing studies in children are limited by lack of random assignment to environment, their reliance on subjective parent-report measures of attention, and the use of proxy measures that indirectly assess endogenous and/or exogenous attention (e.g., academic performance) (Kaplan, 1995; Taylor et al., 2002; Wells and Evans, 2003; Kuo and Faber Taylor, 2004). No studies to date have used an experimental design and objective measures of attention to examine the impact of time spent in natural environments on typically developing children’s attention. Moreover, no previous study has explicitly examined both endogenous and exogenous modes of attention in children in contrasting urban and natural environments.

The distinction between endogenous and exogenous attention is central to ART, yet at the time that this study was initiated, there were no tools available to robustly measure both modes of attention in a unified framework. Furthermore, modern perspectives on attention (Klein and Lawrence, 2012) delineate not merely the modes of attention but also their domain, including temporal, spatial, and task. While tests such as the Attention Network Test and its derivatives (ANT-R, ANT-I, and others) are popular in studies that seek to measure attention across its various forms to evaluate specific effects of an intervention, these tests have deficiencies in their design that prompted us to develop an improved test of attention. Specifically, the original ANT failed to achieve orthogonal manipulation of temporal and spatial attention, making it impossible to evaluate their interaction. The original ANT also fully confounded exogenous and endogenous forms of both spatial and temporal attention. The ANT-I is a test that achieved orthogonal manipulation of temporal and spatial attention, and eliminated the confound between exogenous and endogenous spatial attention, but with the latter achieved by focusing on exogenous spatial attention alone, and with no attempt to eliminate the confound between exogenous and endogenous temporal attention.

The Combined Attention Systems Test (CAST) was developed as an improvement on similar tasks, including the Attention Network Test (ANT) and the Attention Network Test-Interaction (ANT-I). The ANT was employed in a prior study that examined nature exposure (although only images presented in a laboratory) and attention (Berman et al., 2008). Like the ANT, the CAST is a computerized measure of the three attentional networks (alerting, orienting, and executive attention), and is a sensitive and theoretically driven measure. The CAST was employed in the current study because it addresses known limitations of the ANT and ANT-I and because it separately measures exogenous and endogenous attention, and executive attention, which are central to examining the Attention Restoration Theory (MacLeod et al., 2010; Lawrence et al., 2011).

The aim of the present study was to test attentional changes, pre- and post-exposure to nature compared to an urban environment in a sample of typically developing children and adolescents, using a quasi-experimental design. Advancements in the area of attention task development were used to more robustly examine how exposure to nature affects both exogenous and endogenous attention performance in children. In order to assess the impact of exposure to nature on both exogenous and endogenous attention, we employed the CAST (Lawrence et al., 2011; Lawrence, 2018) to parse out exogenous and endogenous modes of attention. More specifically, we employed a between-subjects pre/post design, wherein we assessed children’s exogenous and endogenous attention performance before and after exposure to either a natural environment or an urban environment. Consistent with ART, we hypothesized that children who were exposed to natural environments during a 30-min reflective walk would demonstrate specific improvements in endogenous attention, as indexed by change in performance on the CAST. Further, given that ART suggests that the restorative potential of natural environments lies in their ability to engage exogenous attention in lieu of endogenous attention (Kaplan, 1995), we hypothesized that exposure to nature would only improve endogenous attention, and thus did not expect to observe changes in participants’ exogenous attention. Finally, consistent with ART and the body of literature which suggests that urban environments are non-restorative (Herzog et al., 1997; MacLeod et al., 2010; Li et al., 2011), we did not expect changes in endogenous or exogenous attention in those children and adolescents assigned to a 30-min reflective walk through an urban environment. The current study is an important first step in exploring exposure to nature as a potential remediation for attentional fatigue in children.

## Materials and Methods

### Participants

Ninety children participants aged 8–15 years were recruited over a 1-year period through community bulletins, newsletters, paid advertisements, and emails distributed to families that had previously participated in studies in our laboratory. The children were initially assessed for eligibility for enrollment in the study and 19 were excluded. Children were required to have an estimated IQ equal or greater than 80, normal or corrected-to-normal vision, no history of psychiatric/psychological diagnoses, no history of severe head injury, and no significant neurological disorders affecting the central nervous system. The study received ethical approval from Dalhousie University’s Institutional Research Ethics Board in August 2012. However, registration of the study as a clinical trial occurred retrospectively as there was no determination during ethics review that the study fit the definition of a trial. The authors confirm that all ongoing and related trials for this intervention are registered.

Participants received $15 as an honorarium for study participation, and parents were entered to win one of two $50 gift cards to a bookstore as compensation for their time. A total of 71 children were assigned to one of two conditions: an urban walk or nature walk. The logistics of testing outside of a laboratory made true random assignment impossible for this study. However, participants blindly assigned themselves to study condition; participants were informed that there were two possible locations to which they could be assigned and then were asked to select a participation date, following which the testing location prescheduled for that date was revealed.

**Figure 1:**
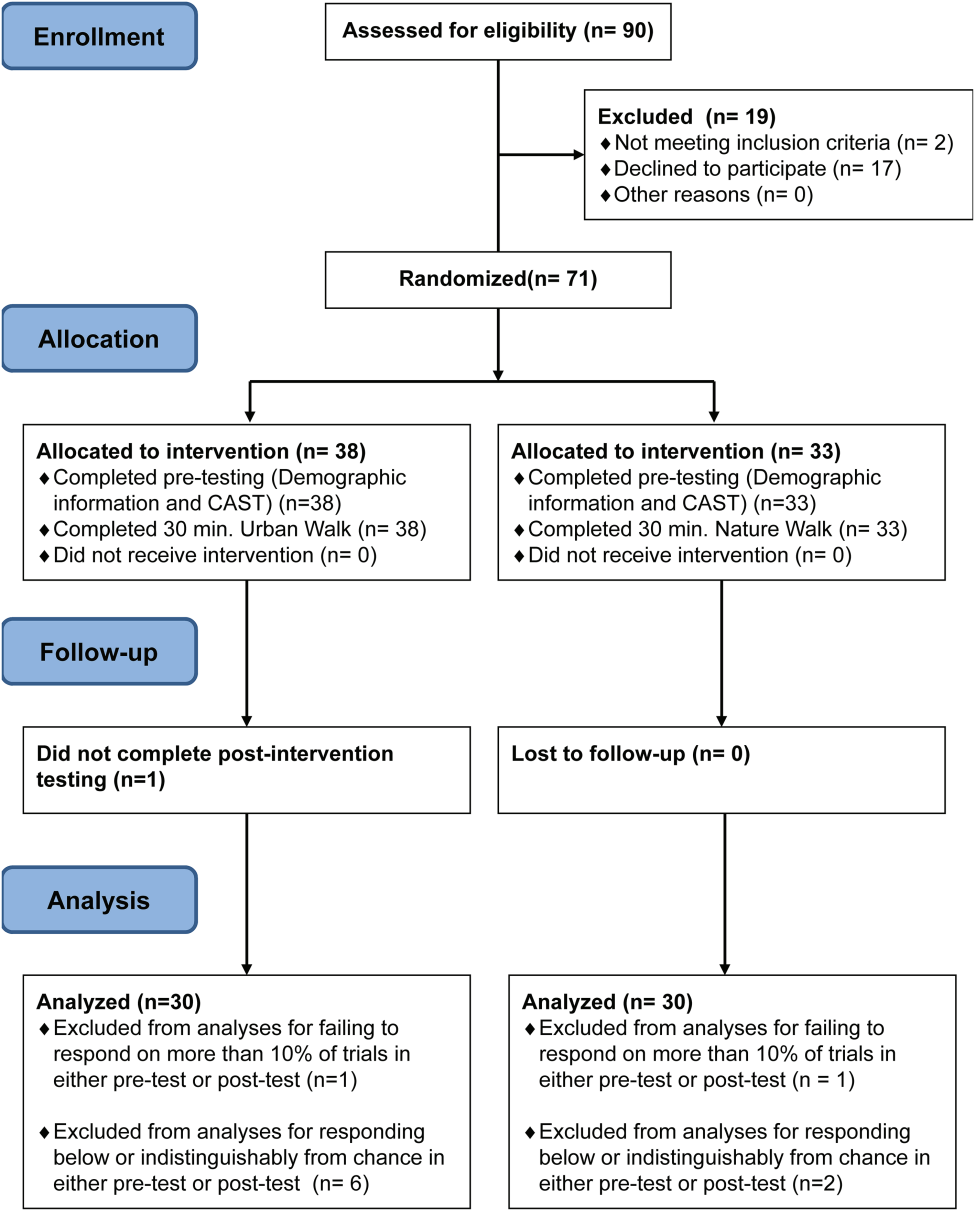
CONSORT (CONsolidated Standards of Reporting Trials) flow diagram.

Thirty-eight children (42% male) participated in the urban condition and 33 children (45% male) participated in the nature condition. Participants were primarily Caucasian (82.6%). One participant in the urban group discontinued the study before completing the second session. Of the 70 participants that completed the study, the CAST data of 10 participants were excluded due to either performing at chance during one of the sessions or failing to respond on more than 30% of trials. Thus, the analyses for the CAST were conducted on a sample of 60 participants [nature condition, *n* = 30 (15 female); urban condition, *n* = 30 (16 female)] (Figure 1). Bayesian regressions indicated that the groups were well-matched (no differences) in terms of key demographic features, study features, and characterization measures (Table 1). No significant group differences were evident for level of parental education or household income.

**Table 1:** Study sample characteristics.

	Overall (*n* = 60; 29 M/31F)		Nature (*n* = 30; 15 M/15F)		Urban (*n* = 30; 14 M/16F)		Difference^*^
	*μ*	*σ_x̅_*	*μ*	*σ_x̅_*	*μ*	*σ_x̅_*	
Age (years)	11.4	2.3	11.3	2.3	11.5	2.3	0.4 (−0.7–1.5)
WASI Full IQ	115.1	11.7	114.2	10.9	116.0	12.6	1.5 (−4.4–7.7)
CNS	4.9	0.9	4.8	1.0	4.9	0.9	0.1 (−0.4–0.5)

M, male; F, female; WASI, Wechsler Abbreviated Scale of Intelligence, standard scores are reported; CNS, Connectedness to Nature Scale, raw scores are reported.

* *Difference, posterior median and 95% Credible Interval (CrI)*.

### Characterization Measures

A Demographic and History Questionnaire, an IQ test, and the Nature Connectedness Scale (CNS) (Mayer and Frantz, 2004) were administered in order to characterize the participants and facilitate comparison of participants in the two groups. Participants’ parents/guardians completed a demographic and history questionnaire assessing information pertaining to child characteristics (e.g., age, birth date, and ethnicity), family circumstance/composition, child and family psychiatric history, and current medications. The Wechsler Abbreviated Scale of Intelligence (WASI) (Wechsler, 1999) was administered to provide an estimate of participant’s intellectual ability, in order to ensure that participants met inclusion criteria (IQ ≥ 80). The WASI was not administered to individuals who provided consent for the researchers to obtain the results of IQ testing that had been conducted in another clinical or research setting within the past 2 years. Each child completed a 10-item modified version of the original CNS scale (Mayer and Frantz, 2004) developed by the scale’s creators. The child version uses a 7-point scale (strong disagree to strongly agree). Higher scores indicate stronger connectedness to nature (child range = 10–70). *Combined Attention System Test* (*CAST*) (Lawrence, 2018). The CAST is a game-like computerized measure designed to assess and isolate both endogenous and exogenous attention. Accordingly, the CAST is composed of two separate tasks: the endogenous task and the exogenous task.

### Procedure

**Figure 2:**
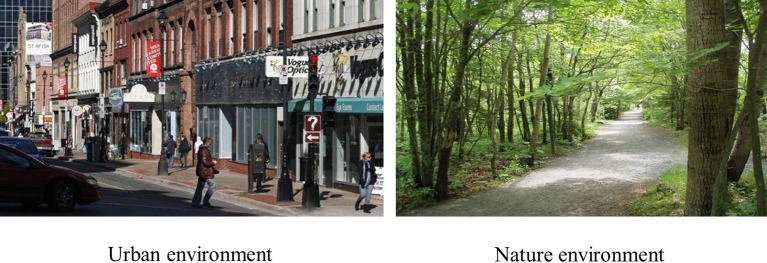
Photos of the urban and natural exposure environments.

For the purposes of this study, Shubie Park in Dartmouth, Nova Scotia (geometric center: 44.70°N, −63.55°E) was selected as the location for exposure to a natural environment and a busy section of downtown Halifax, Nova Scotia (geometric center: 44.64°N, −63.57°E) was selected for exposure to an urban environment (Figure 2). These locations were chosen due to their proximity to buildings available for the testing phase of the study that provided easy and direct access to urban and natural environments.

Children were placed into groups (between two and four children) and were then led on a 30-min walk through either the nature or the urban exposure environment. The walks consisted of a 2-km (approximately 1.3 mile) route for each exposure condition and the distance was objectively confirmed using a GPS data logger. Pre/post exposure testing took place at indoor locations within 5 min of the walk locations. WASI administration took place in the lab of Dr. Shannon Johnson, during a separate testing session. Walks and corresponding data collection took place between the months of July and November over a 2-year period.

Prior to study participation, a parent/guardian of potential participants completed a brief phone-based screening interview to determine if their child(ren) met preliminary inclusion criteria (note: if cognitive testing had not been conducted previously, it was not possible to determine if participants met IQ cut-off prior to study administration). If preliminary inclusion criteria were met, we explained that participation in this study involved blind assignment to one of the two locations to minimize expectancy biases (i.e., participants were not aware of the specifics regarding the testing location they were not assigned to). The researchers then provided the parent/guardian with several possible testing dates, and asked them to select a date and time during which their child(ren) could complete the study. After the date and time were selected, the parent/guardians were provided with location. Parents were provided with the *demographic and history questionnaire* in advance, and given the option of bringing the completed forms with them on the day of the study, or completing the forms while their child(ren) participated.

Participants completed the study in groups of 1–4 (*M* = 3.34). At least two research assistants were present at all times to provide instructions, address any questions, and monitor for compliance. Upon arrival, research assistants obtained assent for study participation from the children and consent from a parent/guardian. Researchers then administered the *Connectedness to Nature Scale*, followed by the CAST to each participant. All participants completed the questionnaire and attention testing at the same time (i.e., once the researchers provided each child with task instructions, the children were prompted to begin the tests). Accordingly, both testing locations were able to accommodate several workstations. Each child was seated so that they could not see the other participants during the task. Following completion of the CAST, participants were provided with water, and instructions regarding their 30-min walk. They were encouraged to allow the research assistant to set the pace for the walk, to take notice of their surroundings, and to keep social interactions to a minimum. After the walk, participants completed the second administration of the CAST.

In total, the endogenous and exogenous tasks required approximately 26 min to complete. Administrations of the CAST were completed on Macbook laptops, with 28.5-cm screens, running on Lion OS X operating software. Participants sat approximately 64 cm from the screen and were provided with game controllers and audio headphones. The headphones were calibrated using a Class 2 sound level meter to between 60 and 80 decibels for all tones. For both tasks, participants were to indicate, as quickly as possible, the direction that a target fish (subtending 2° of visual angle) is facing by pressing either the right- or left-hand trigger button. Speed of response is emphasized more than accuracy, although both are encouraged as part of the instructions. The target fish appears against a white background, facing left or right, 5° degrees to the left or right of the central fixation. The target fish is presented alone or surrounded by a school of fish (one above, one to the right, one below, one to the left; spaced 0.2° from the target fish) that are facing in either the same or opposite direction of the target fish.

The variables manipulated in the task are target fish direction (left vs. right facing), target fish location (left vs. right), flankers (none, congruent, incongruent), auditory stimuli presented bilaterally (endogenous task: background noise change vs. background noise held constant; exogenous task: background noise volume increase vs. background noise volume held constant), and visual cues (endogenous task: central arrow valid vs. central arrow invalid; exogenous task: valid peripheral black dot vs. invalid peripheral black dot). All possible combinations of these variables yield 72 trial types in the endogenous task, and 48 trial types for exogenous task. For each task, there is a practice block of 24 randomly chosen trials, followed by two experimental blocks, wherein all trial types, respective to each task, are presented at random. Thus, in total participants complete 48 practice trials and 240 experimental trials.

The order of task-presentation was counterbalanced across participants in the urban and nature groups, and there was no group difference for the order of presentation [*χ*2 (1, *N* = 40) 0.04, *p* = 0.85]. The manipulated variables across the two tasks allow for the measurement of a number of dependent variables: (1) exogenous alerting network score, (2) exogenous orienting network score, (3) endogenous alerting network score, (4) endogenous orienting network score, and (5) two measures of the executive network of attention.

Please refer to Supplementary Material for a detailed description of the attention measures and CAST procedure.

### Data Pre-processing and Modeling

The collected data were pre-processed to remove: all trials on which participants failed to make a response (0.1% of trials); all trials on which responses were made prior to target appearance (0.6% of trials); and all trials on which response times were faster than 200 ms (0.3% of trials). The latter exclusion criterion was determined by prior experience with RT data suggesting that responses faster than 200 ms tend to be anticipatory responses unrelated to target processing, as well as application of a generalized additive model of trial accuracies predicted by trial RTs, showing that only above about 200 ms do responses rise above chance performance.

Bayesian inference was achieved using the Stan (Stan Development Team, 2017) probabilistic programming language *via* the RStan package for R (R Core Team, 2017). Response time and accuracy from both subtests were modeled simultaneously, where trial-by-trial accuracy was modeled as a binomial event and trial-by-trial response time was modeled as having log-normal measurement noise. Within a given participant, the influence of the manipulated variables on accuracy was modeled as affecting the log-odds of error while their influence on the response time was modeled as affecting the log-mean response time; the scale of the log-normal measurement noise was also modeled for each participant. The full set of coefficients relating a given participant to their trial-level data was modeled as varying across participants through a multivariate normal distribution in a hierarchical model that sought inference on the population-level coefficient means, variabilities, and correlations. Notably, as compared to more traditional approaches to data analysis (e.g., ANOVA) that would employ independent analyses of response time and accuracy data, by modeling the response time and accuracy data in the same model, we achieve more accurate and informed inference on their associated coefficients at both the participant and population level to the degree that there are correlations among them manifest in the population, which is a strong expectation for these measures (for example, slower participants tend to be more accurate; participants with larger flanker effects on response time tend to have larger flanker effects on response accuracy). In the terminology of De Boek and Minjeong (De Boeck and Jeon, 2019), this is a joint hierarchical model and reflects an approach to the analysis of timed tests that is now relatively common in the psychometric literature (Van Breukelen, 2005; van der Linden, 2007; Loeys et al., 2011) but has yet to see widespread adoption in cognitive psychology (c.f., Molenaar et al., 2015). Independent and weakly informed priors were used for all population-level parameters.

Data, analysis code, and summary tables of response times by task are available online *via* the Open Science Framework (OSF) website (Rainham and Lawrence, 2019).

## Results

The results are reported below in two sections. In the first section, we report the main effects of the attentional measures, validate an absence of group differences on these measures at session 1, and report interactions between these measures and session (likely due to learning). In the second section, we test our hypotheses by examining the three-way interactions between group, session, and attention measures. It is amid these three-way interactions that we would expect to find support for the Attention Restoration hypothesis.

### Validation of Combined Attention Systems Test, Baseline, and Practice Effects

#### Validation of Attention Measures

**Figure 3:**
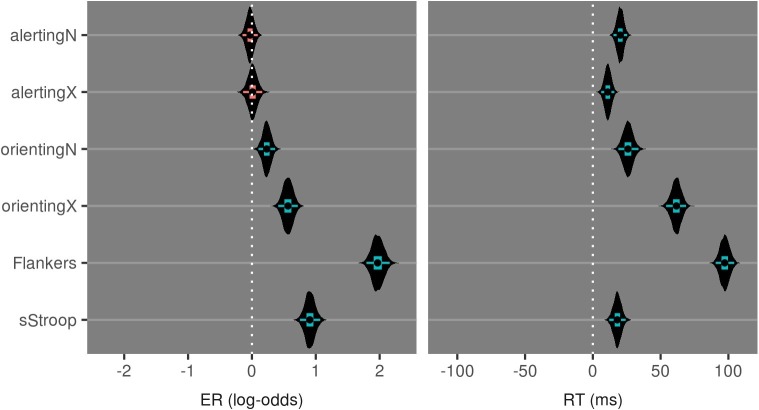
Violin plots of posterior distributions for attentional measures of the CAST. Inner boxplots convey the 95% CrI, 50% CrI, and median value. Coloring of the intervals indicates inclusion of zero in their ranges.

Collapsing across sessions and groups, Figure 3 and Table 2 indicate that all attention measures of the CAST are observed with credibly non-zero magnitude except the endogenous and exogenous alerting effects on error rate. These findings indicate that the task manipulations within the CAST yielded the intended effects for both the endogenous and exogenous tasks.

**Table 2:** Median and 95%CrI values from posterior distributions for attentional measures of the CAST.

Effect	ER (log-odds)	RT (ms)
alertingN	0.0 (−0.1:0.1)	20 (15:25)
alertingX	0.0 (−0.1:0.2)	11 (6:16)
orientingN	0.2 (0.1:0.4)	26 (18:33)
orientingX	0.6 (0.4:0.7)	62 (54:69)
Flankers	2.0 (1.8:2.2)	97 (91:104)
sStroop	0.9 (0.7:1.1)	18 (12:24)

N, endogenous task; X, exogenous task.

#### Validation of Absence of Group Differences at Session 1

**Figure 4:**
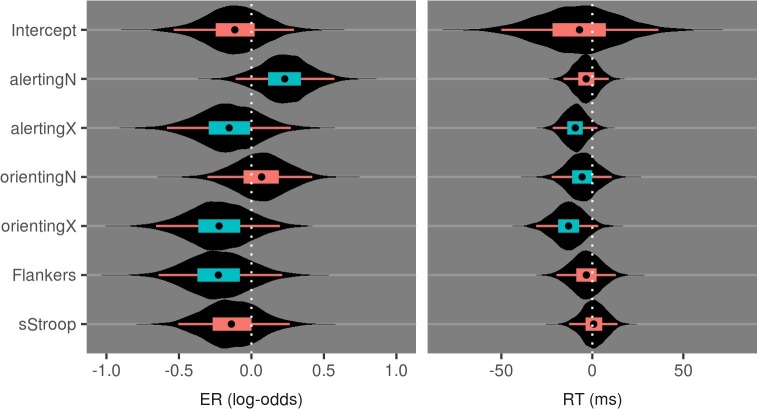
Violin plots of posterior distributions for the effect of Group in Session 1 attention measures of the CAST. Inner boxplots convey the 95% CrI, 50% CrI, and median value. Coloring of the intervals indicates inclusion of zero in their ranges.

Figure 4 and Table 3 show the data from evaluating the effect of group on the attention measures during session 1. Zero falls within the 95% credible interval for all effects, indicating no baseline differences on the CAST between the urban and nature groups for both the endogenous and exogenous tasks.

**Table 3:** Median and 95%CrI values from posterior distributions for group differences at session 1 on attention measures of the CAST.

Effect	ER (log-odds)	RT (ms)
Intercept × Group	−0.1 (−0.5:0.3)	−8 (−51:34)
alertingN × Group	0.2 (−0.1:0.6)	−3 (−16:9)
alertingX × Group	−0.2 (−0.6:0.3)	−9 (−22:3)
orientingN × Group	0.1 (−0.3:0.4)	−6 (−24:11)
orientingX × Group	−0.2 (−0.7:0.2)	−14 (−31:5)
Flankers × Group	−0.2 (−0.6:0.2)	−3 (−20:13)
sStroop × Group	−0.1 (−0.5:0.3)	1 (−12:14)

N, endogenous task; X, exogenous task.

#### Attention Measures and Interactions With Session

**Figure 5:**
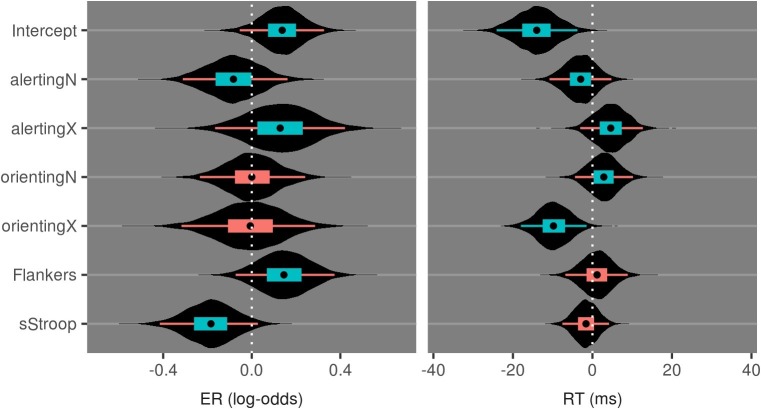
Violin plots of posterior distributions for the effect of Session on attention measure of the CAST. Inner boxplots convey the 95% CrI, 50% CrI, and median value.

Figure 5 and Table 4 show data resulting from the evaluation of the effect of session and its interaction with the attention measures of the CAST. For most effects, zero remains a relatively credible value for the effect of session with the exception of the RT Intercept, reflecting responses that are about 14 ms faster in the second session, and the exogenous orienting effect, reflecting a reduction of about 10 ms in the magnitude of this effect from session 1 to 2. The results demonstrate some minimal practice effects, which is consistent with many cognitive tasks.

**Table 4:** Median and 95%CrI values from posterior distributions for the effect of session on attention measures of the CAST.

Effect	ER (log-odds)	RT (ms)
Intercept × Session	0.1 (−0.1:0.3)	−14 (−24:−4)
alertingN × Session	−0.1 (−0.3:0.2)	−3 (−11:5)
alertingX × Session	0.1 (−0.2:0.4)	5 (−3:13)
orientingN × Session	0.0 (−0.2:0.2)	3 (−5:10)
orientingX × Session	0.0 (−0.3:0.3)	−10 (−18:−1)
Flankers × Session	0.1 (−0.1:0.4)	1 (−7:9)
sStroop × Session	−0.2 (−0.4:0)	−2 (−7:4)

N, endogenous task; X, exogenous task.

### Influence of Exposure Conditions

**Figure 6:**
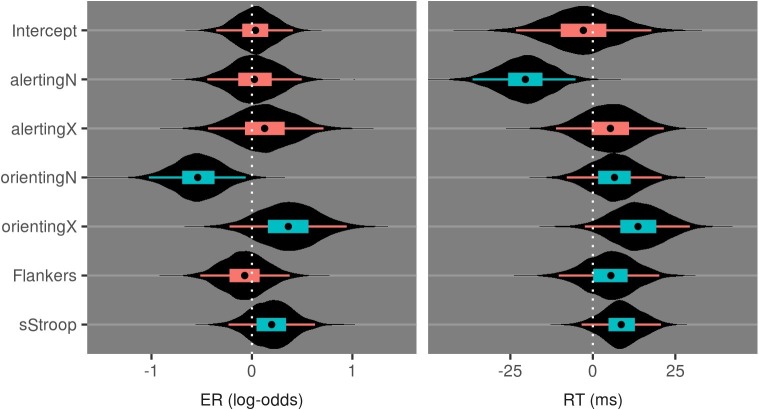
Violin plots of posterior distributions for the effect of the Session-by-Group interaction on attention measures of the CAST. Inner boxplots convey the 95% CrI, 50% CrI, and median value.

**Figure 7:**
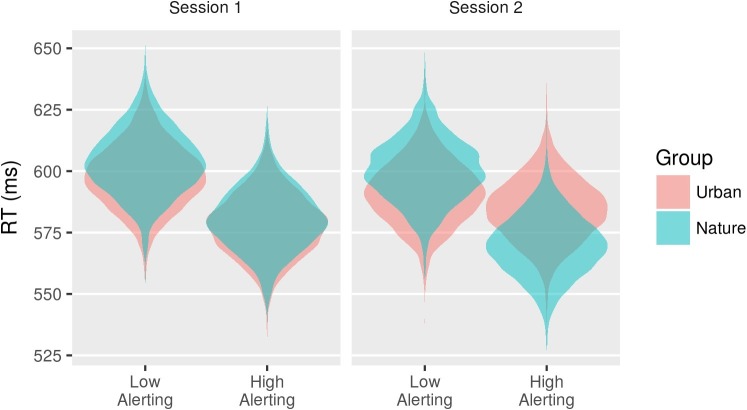
Violin plots of the posterior distributions for the conditions associated with the interaction between Session, Group, and the AlertingN effect on RT.

**Figure 8:**
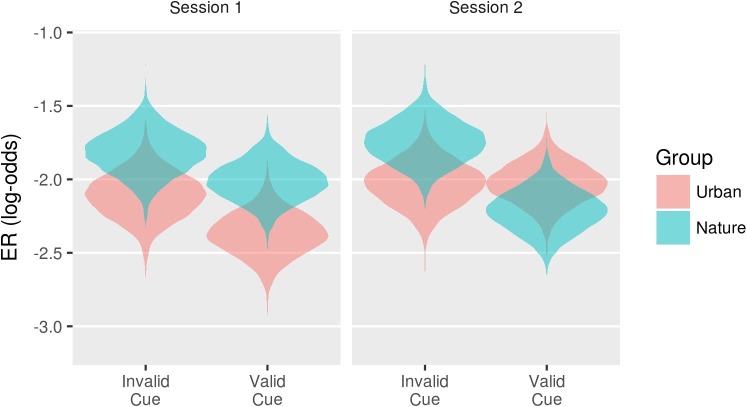
Violin plots of the posterior distributions for the conditions associated with the interaction between Session, Group, and the OrientingN effect on ER.

Figure 6 and Table 5 show the posterior distributions for the coefficients reflecting the three-way interaction between group, session, and each effect of attention measured by the CAST. Zero remains a relatively credible value for all except the endogenous alerting effect and the endogenous orienting effect. As shown in Figure 7, at baseline, the two groups demonstrated relatively similar endogenous alerting in RT. Compared to baseline, the urban group’s endogenous alerting was reduced at session 2, driven by both “high and low” alerting conditions moving toward the mean. In contrast, the nature group’s endogenous alerting increased at session 2 compared to baseline, driven primarily by reduction of RT: *d* = 0.85 (95% CI: 0.21–1.8) in the “high alerting” condition. As shown in Figure 8, at baseline, the two groups demonstrated relatively similar error rates (ERs) for endogenous orienting. Compared to baseline, the urban group’s endogenous orienting was reduced at session 2, driven by an increase in ER in both conditions with a larger increase in the “valid cue” condition. In contrast, the nature group’s endogenous orienting increased at session 2 compared to baseline, driven primarily by a reduction of ER: *d* = 1.45 (95% CI: 0.17–7.18), in the “valid cue” condition. These patterns in endogenous alerting and endogenous orienting are consistent with a fatigued endogenous attention system in the urban group and a refreshed (or even enhanced) endogenous attention system in the nature group. In addition to the R code and study data, descriptive statistics (mean and cross-standard deviations) in each condition (urban or nature) for RT, log-RT, and the error rate are provided as Supplementary Material.

**Table 5:** Median and 95%CrI values from posterior distributions for the effect of the Session-by-Group interaction on phenomena of attention measured by the CAST.

Effect	ER (log-odds)	RT (ms)
Intercept × Session × Group	0.0 (−0.3:0.4)	−3 (−23:18)
alertingN × Session × Group	0.0 (−0.4:0.5)	−20 (−36:−5)
alertingX × Session × Group	0.1 (−0.5:0.7)	5 (−10:21)
orientingN × Session × Group	−0.5 (−1.0:−0.1)	7 (−8:22)
orientingX × Session × Group	0.4 (−0.2:0.9)	14 (−2:30)
Flankers × Session × Group	−0.1 (−0.5:0.4)	5 (−9:20)
sStroop × Session × Group	0.2 (−0.2:0.6)	9 (−3:21)

N, endogenous task; X, exogenous task.

## Discussion

The primary goal of this study was to test the hypotheses set forth by ART, that exposure to nature is restorative to attention, in a sample of typically developing children and adolescents. As hypothesized, we found that children in the nature exposure condition demonstrated slight improvements in endogenous attention and no change in exogenous attention performance following the walk. Finally, with regard to the third hypothesis, we found that there was no improvement in the urban group, but that counter to our expectations, performance on two endogenous attentional measures worsened following exposure.

We examined the following three hypotheses: (1) Children who were exposed to a natural environment (i.e., path through a forest) during a 30-min reflective walk were expected to demonstrate improvements in endogenous attention, as indexed by change in performance on the CAST. (2) Exposure to nature was not expected to improve participants’ performance on the exogenous conditions of the CAST. (3) No changes were expected in endogenous or exogenous attention performance in those children and adolescents assigned to a 30-min reflective walk through an urban environment.

There is some evidence to support our first hypothesis, improvement in the nature group following their walk. However, this was not solely due to better performance in the nature group. We found effects differed between the urban and nature groups for endogenous alerting and orienting, which were due to both improved attentional performance in the nature group and worse performance in the urban group following the interventions.

Our findings are consistent with similar experimental studies that interpreted group differences on the ANT executive measure as evidence for improvements for the nature exposure group. Similarly, we found that the urban group demonstrated worse performance post-exposure relative to pre-exposure. Previous studies have also examined and reported significant results on the influence of nature contact on several components of cognitive function (Herzog et al., 2003; Hartig and Staats, 2006; Berman et al., 2008); however, these studies typically employ measures of attention and cognitive function which do not necessarily align with the attentional constructs proposed in ART. That is, previous studies have not been able to tease apart exogenous and endogenous attention, but have instead identified changes in other constructs, such as working memory. Importantly, the current study is the first to differentiate the effects of exposure to nature on exogenous versus endogenous attention.

An important contribution of this study is the application of the CAST, a novel attentional task that has the ability to separate exogenous from endogenous attentional measures. This task allowed us to directly test the core tenets of ART, which previous studies were unable to do given the use of less sophisticated measures of attention. Using the CAST, in conjunction with a data analysis approach that allowed us to model response time and error rates simultaneously, we have identified that not only does nature lead to some improvements in endogenous attentional performance, but importantly spending time in urban environments has a detrimental effect on endogenous attention. This is consistent with ART as urban environments would be expected to lead to attentional fatigue and exposure to nature can serve as a buffer from attentionally fatiguing environments. We replicated the methodology of previous studies, but were able to address specific questions by employing the CAST, and found that the key hypotheses of ART were supported.

However, differences in attention task performance between the nature and urban groups may also have arisen due to inherent features of the study approach. For example, group differences may be due to attenuation of attentional control or performance instability between attention tasks pre- and post-exposure to either the urban or nature conditions. Although not explored here, there is a possibility that improvement in attentional performance arising from restoration (*via* contact with nature) or depletion (from exposure to urban environments) originates from specific locations within the response time distribution, and thus it is the control of attention regulating differences between groups (Kaplan and Berman, 2010). We are unable to exclude the potential influence of attention task position among all tasks during administration of the CAST. Reversing the order of tasks among participants as part of a counterbalanced design may provide additional insight into the importance of task order in administration of the CAST. In addition, we did not conduct sensitivity analyses of the impact of model selection as the priors employed were weakly informed. Readers are welcome to assess stability in intra-individual attentional performance using the study data available from the repository.

Our findings support the growing body of literature that indicates that spending time in nature is beneficial for children. Correlational studies have suggested improved attentional functioning in children who experience more time in natural settings or in urban settings that include more natural features. The current study provides further evidence for improvements in endogenous attention using a controlled design and a short exposure duration. According to ART, exposure to nature may restore endogenous attention through influence on exogenous attention which can transiently interrupt goal-directed behavior. Exogenous cues in natural environments, such as changes in luminance, the complexity of shapes and patterning (arousing fascination), or peripheral cues arising from the movement of animals, insects, or the movement of vegetation from wind, are components of natural environments that contribute to restoration or arousal *via* interruption of endogenous or intentional, directed attentional resources. Our results indicate that even a relatively short exposure to nature (30–40 min) improves endogenous attention and a relatively short dose of an urban environment taxes endogenous attention in children. Thus, these findings suggest that increasing time in nature, while also considering ways to buffer the detrimental effects of urban exposure, is necessary for maximizing children’s attention.

Taken together, the findings of this study offer some important considerations for future studies examining the benefits of nature exposure for children. For example, our analysis reveals only a modest improvement to endogenous attention following exposure to nature. We are reasonably confident that this was not due to the quality of the exposure, nor to the sensitivity of our attention measure. The nature walk, and associated data collection, was completed within a heavily forested urban park that is 40 ha in area with natural walking paths and various natural water features (canal, lakes). We chose this site because there are no features of urban development or other urban stimuli once on the trails. In addition, the CAST is a theoretically driven and sensitive measure of a variety of exogenous and endogenous attentional processes. Compared to prior studies (e.g., Berman et al., 2008; Bratman et al., 2012), the walk environments and the attention measure were improvements in study design in terms of quality of exposure and measurement of attention.

Although we do not question the quality of the nature exposure, we have reservations about whether the amount of time spent in nature was sufficient to lead to a dramatic improvement in attentional performance in children. Children may require more exposure to obtain similar levels of improvement as adults. The question of how much time children should spend in nature is important. How much time would be enough to invoke a change in attention? It is possible that one experience, regardless of duration, would not be enough. Children may require repeated experiences in order to develop greater familiarity with natural environments, and acquire improved attention as a result of spending time in nature.

When considering the effects of nature exposure on children’s attention, it is also important to consider intergenerational differences in nature experience. The idea that historically humans have spent more than 90% of time in nature (Bratman et al., 2012) provides an evolutionary basis from which to conclude that we are pre-inclined to feel comfortable in and innately connected with nature. However, today’s children spend, on average, less than 60 min outdoors each day, usually as part of a trip from one location to another (The David Suzuki Foundation, 2012). As children spend more time indoors, they become increasingly disconnected from nature and the ecology of the outdoors. Perhaps, as a function of becoming more disconnected from nature, children do not experience or perceive nature to be restorative in the way that research suggests adults do (Hartig and Staats, 2006). For adults, spending time in nature creates connections to learned experiences about how they feel in this type of setting. Individuals with existing connections to nature, and who may have already experienced restorative effects, may require a much smaller dose of time in nature to reap benefit. For example, study participants in Berman’s study (Berman et al., 2008), with average age of 23 years, may have spent more time developing connections to nature in the late 1980s/early 1990s. In addition to spending much less time in nature, due to significant increases in the allocation of time to indoor activities (e.g., computing, television), children may develop a fear of natural settings (Louv, 2005). If children have become so disconnected from nature that they are afraid of or uncomfortable in nature, it seems unlikely that spending time in nature would provide them with the same rewarding benefits that it provides adults. While this theory points toward compelling empirical questions, to date, there have been few studies of the potential negative impacts of time spent in nature. Regardless, potential generational differences in nature contact may afford an opportunity for additional research focused on the quantity and quality of children’s exposure to nature as a determinant of cognitive benefit.

In addition to familiarity with and the time spent in natural environments, the presence of unfamiliar children in the exposure conditions and dehydration are additional factors to consider. The design of the current study allocated children to small groups, primarily for practical reasons such as cost associated with space rental and scheduling. In similar studies evaluating nature’s restorative potential, participants were exposed and evaluated independently (Berto, 2005; Berman et al., 2008). The presence of unfamiliar children may limit the restorative potential of time spent walking in nature, possibly through stress or the direction of attention to navigating social position and interaction. Potential for the creation of discomfort through socialization stressors in the context of nature may limit the restorative potential of nature contact. We recommend future research designs that include independent nature-based experiences, possibly with a member of the research team as a guide. Group-based exposure designs should include a component to evaluate relative comfort associated with participation in the group, in addition to feelings associated with immersion in natural environments, as well as the inclusion of participants with existing relationships such as with friends or family. In addition to social influences, research has shown that children can be at risk of mild voluntary dehydration, where changes in hydration status have been linked to cognitive performance (Fadda et al., 2012). Although we did not monitor water consumption, participants’ water intake during the study was not different from their usual drinking habits (one glass of water or less). Since participants did not change their nutritional and physical activity habits prior to or during the study, it is unlikely that the amount of water consumed, if any, significantly affected levels of hydration.

Although there is some evidence to support that attention changes following exposure to nature, it is possible that attention is not the ideal outcome measure for studies of nature exposure in children. The ways in which children (particularly children of different ages) and adults experience, interact, and behave in nature are likely quite different. The utility of nature in early childhood (3–6 years of age) is to satisfy a child’s own personal material or physical needs, and to provide an environment in which to achieve feelings of control, security, or comfort. Children in early childhood may display affection, indifference, or even anxiety when in contact with natural environments or other species. As children age (from 6–12 years of age), they develop an increased curiosity and capacity for assimilating knowledge and understanding of natural environment. This period of middle childhood integrates the natural world as a place for exploration and discovery and the establishment of self-identity (as different from parents and siblings). Natural environments become a medium that children use to construct their own secret places (e.g., forts, hiding places), where play occurs and stories are created and re-enacted. Nature at this stage may be restorative in the sense of affirming capacity (e.g., self-efficacy) and in the creation of long-term memories or attachments to natural environments and materials.

In adolescence, the concept of nature becomes more abstract, particularly as children learn about ecological function, values, and stewardship. However, nature also becomes a context within which to engage in daring and challenging activities that test and nurture self-confidence and identity. For example, adolescent participants of outdoor camps and leadership schools report that wilderness experiences improve capacity to function in urban settings, and result in greater appreciation for natural environments (Kellert and Derr, 1998). Compared to urban environments, natural environments are inherently unstable, unpredictable, and challenging and require continued alertness and attention. Children’s play in and exploration of nature may tax their endogenous attention and increase arousal, all the while increasing other abilities such as creativity and sensory processing. Over repeated exposures, the time children spend in nature would be expected to lead to comfort in and connections with nature that yield the positive benefits consistent with ART. In addition to being more sensitive to the relationship between children’s age and the kinds of experiences they may have in nature, it would seem prudent to explore and measure children’s affective responses, behaviors, and connectedness with nature following their time spent in natural environments, as well as longer term outcomes such as learning, creativity, and academic performance.

## Conclusion

This study investigated whether exposure to nature resulted in improvements to endogenous attention. Using a quasi-experimental design with random assignment, the performance of typically developing children and adolescents on a state-of-the-art attention task was evaluated before and after exposure to a nature or urban condition. We found differences between the exposure groups on two endogenous attentional measures due to both improved performance in the nature group and worse performance in the urban group following exposure. We argue that the unexpected detrimental effects on endogenous attention in the urban group are consistent with ART in that urban exposure fatigues attention. Several explanations are provided as to why the effects in the nature group were less striking than we anticipated, including: immersion time in exposure environments, intergenerational effects, familiarity with nature, the influence of social interaction during exposure, and the sensitivity of attention in children to environmental influences. Exposure to nature in childhood is important for so many reasons other than influences on attention. The results of this work should not only be used to support efforts to increase time children spend in nature, but to also consider ways to buffer the potential negative effects of urban exposure. This is particularly important given the trend toward urbanization. Further research is necessary to address some of the limitations described here, and to understand the effects of exposure to natural settings on children’s development in a variety of important domains.

## Data Availability Statement

Data, analysis code (STAN) and summary tables of response times by task are available online *via* the Open Science Framework (OSF) website: https://osf.io/52aqt/.

## Ethics Statement

The studies involving human participants were reviewed and approved by Social Sciences and Humanities Research Ethics Board, Dalhousie University (2012–2698). Written informed consent to participate in this study was provided by the participants’ legal guardian/next of kin.

## Author Contributions

SJ and DR conceived of the research and the study design, and planned for the acquisition, analysis and interpretation of the data. SS participated in data collection, preliminary analysis, and early drafts of the manuscript. ML made substantial contributions to the analysis and interpretation of the data and revisions of the manuscript. All authors made substantive contributions to the development and revisions of the manuscript. SJ and DR are accountable for all aspects of the work and are responsible for ensuring accuracy and integrity of the work.

### Conflict of Interest

The authors declare that the research was conducted in the absence of any commercial or financial relationships that could be construed as a potential conflict of interest.
