# Supplementary material for: Quasi-Randomized Trial of Contact With Nature and Effects on Attention in Children
Source: Front Psychol. 2019 Dec 5;10:2652. doi: 10.3389/fpsyg.2019.02652 (PMC6907393; doi:10.3389/fpsyg.2019.02652)
Supplement: Supplementary file 1 [file Data_Sheet_1.DOCX]

Supplementary Material

# Combined Attention Systems Test (CAST)

All administrations of the CAST were completed on Macbook laptops, with 28.5 cm screens, running on Lion OS X operating system software. Participants were seated approximately 65 cm from the screen. In order to ensure that all four sets of Sony MDRNC7 Noise Cancelling On-Ear Headphones were presenting equivalent auditory stimuli, an Extech instruments Digital Sound Level Meter (RS323, model 407750) was employed to calibrate the sounds played through the headphones. Calibration was completed with the MacBook laptops volumes set to maximum, and the headphones active noise-cancelling feature on. The lowest tone was calibrated to 60 decibels, and the loudest tone was calibrated to 80 decibels.

For both tasks, participants completing the CAST are required to indicate, as quickly as possible, the direction that a target fish (subtending 2° visual angle) is facing by pressing either the right or left hand trigger button on a USB Xbox360 gamepad controller. Speed of response is emphasized more than accuracy, although both are encouraged as part of the instructions. The target fish appears against a white background, facing left or right, 5° degrees to the left or right of the central fixation. The target fish is presented alone or surrounded by a school of fish (one above, one to the right, one below, one to the left; spaced 0.2° from the target fish) that are facing in either the same or opposite direction of the target fish. Figure Supplemental 1 provides examples of target fish presentations (Figure Supplemental 1A and B).

The variables manipulated in the task are target fish direction (left vs. right facing), target fish location (left vs. right), flankers (none, congruent, incongruent), auditory stimuli presented bilaterally (endogenous task: background noise change vs. background noise held constant; exogenous task: background noise volume increase vs. background noise volume held constant), and visual cues (endogenous task: central arrow valid vs. central arrow invalid; exogenous task: valid peripheral black dot vs. invalid peripheral black dot). All possible combinations of these variables yield 72 trial types in the endogenous task, and 48 trial types for the exogenous task. For each task, there is a practice block of 24 randomly chosen trials, followed by 2 experimental blocks, wherein all trial types, respective to each task, are presented at random. Thus, in total participants complete 48 practice trials and 240 experimental trials. In the present study, the order of task-presentation was counterbalanced across participants within both groups. The manipulated variables across the two tasks allow for the measurement of a number of dependent variables:

1) Endogenous Alerting (abbreviated "alertingN"), measured in the "endo" task by comparison of trials on which the warning sound was present versus trials on which the warning sound was absent. It is expected that response times (RTs) will be faster and error rates (ERs) will be lower in the "warning present" trials.

2) Exogenous Alerting (abbreviated "alertingX"), measured in the "exo" task by comparison of trials on which the warning sound was loud versus trials on which the warning sound was quiet. It is expected that RTs will be faster in the "loud warning" trials, but that a speed-accuracy trade-off may also be present such that there will be higher error rates in the "loud warning" trials.

3) Endogenous Orienting (abbreviated "orientingN"), measured in the "endo" task by comparison of trials on which the arrow validly pointed to the location of subsequent target appearance (75% of trials) vs trials on which the arrow invalidly pointed to the location opposite to that of subsequent target appearance (25% of trials). It is expected that RTs will be faster and ERs will be lower in the "valid arrow" trials.

4) Exogenous Orienting (abbreviated "orientingX"), measured in the "endo" task by comparison of trials on which the dot appeared at the location of subsequent target appearance (50% of trials) vs trials on which the dot appeared at the location opposite to that of subsequent target appearance (50% of trials). It is expected that RTs will be faster and ERs will be lower in the "valid dot" trials.

5) Flanker Conflict (abbreviated "Flanker"), measured in both tasks by comparison of trials on which the target fish appears flanked by distractor fish that are oriented in the same direction as the target fish versus trials on which the target fish appears flanked by distractor fish that are oriented in the opposite direction as the target fish.  It is expected that RTs will be faster and ERs will be lower in the "congruent flankers" trials.

6) Spatial Stroop (abbreviated "sStroop"), measured in both tasks by comparison of trials on which the target fish's direction matches its location (i.e. left-pointing fish appearing on the left side of the screen or a right-pointing fish appearing on the right side of the screen) versus trials on which the target fish's direction mis-matches its location (i.e. left-pointing fish appearing on the right side of the screen or a right-pointing fish appearing on the left side of the screen). Because the spatial Stroop effect is known to interact strongly with the flanker effect, the spatial Stroop effect is computed using only those trials without flankers. It is expected that RTs will be faster and ERs will be lower in the "spatial Stroop congruent" trials.

Trials on the endogenous task are presented as follows (see Fig Supplemental 2A): A 60-decibel background noise is played throughout the task. Trials begin with the centrally located presentation of an arrow (pointing left or right; subtending 1**°** the visual angle), contained within an outline of a shape (square or circle; subtending 2**°** the visual angle). This stimulus (i.e., shape-arrow combination) remains on the screen throughout the duration of the trial. The direction of the arrow indicates the probable (accurate 66% of the time) location of the subsequent target (i.e., 5° to the left or right of the central fixation). The shape indicates whether the background noise will change prior to the presentation of the target. In our study, for half of the participants a square indicates the background noise will remain the same and a circle indicates the background noise will change and vice versa for the remaining participants. A non-aging (i.e., random exponential interval) inter stimulus interval (ISI) with a minimum of 1000 ms and a mean of 2000 ms follows the presentation of the shape-arrow combination. At the end of the non-aging ISI, on background noise change trials, the noise changes from mono pink noise (same noise played to both ears) to stereo pink noise (different noise played to each ear) for 100ms, after which the background noise changes back to mono pink noise. A change in background noise provides participants with the information that the target will be presented in 1000 ms. Regardless of background noise change presence/absence the target is presented 1000 ms after the end of the non-aging ISI, and remains on the screen for 1000 ms or until a response is made. To enhance motivation and the game-like nature of the task, feedback is then presented on the screen (subtending 1**°** the visual angle) for 1000ms. When participants provide a response feedback consists of the centrally located display of the participants’ reaction time to target. When no response is provided by the participant feedback consists of the centrally located display of the word “MISS.”

Trials on the exogenous task are presented as follows (see Fig Supplemental 2B): Trials begin with a centrally located presentation of a fixation cross (subtending 1**°** the visual angle) which remains on the screen for the duration of the trial. Presentation of the fixation cross is followed by non-aging (i.e., random exponential interval) inter stimulus interval (ISI) with a minimum of 1000 ms and a mean of 2000 ms. At the end of the non-aging ISI, the background noise changed from mono pink noise (same noise played to both ears) to stereo pink noise (different noise played to each ear) for 100 ms, after which the background noise changed back to mono pink noise. On half the trials the noise remains a constant volume through this change, while on the remaining half of trials the volume increases during this change. Simultaneous with the sound change, a non-predictive peripheral visual cue (i.e., a black circle subtending 2**°** the visual angle, appears either 5° to the left or right of the central fixation) is presented. The target is presented 5° to the left or right of the central fixation, 200 ms after the end of the non-aging ISI, and remains on the screen for 1000 ms or until a response is made. As in the endogenous task, feedback is then presented on the screen for 1000 ms (subtending 1**°** the visual angle). For trials where participants provide a response, feedback consists of the centrally located display of the participants’ reaction time to target (e.g., 557 ms). When no response is provided participant feedback consists of the centrally located display of the word “MISS.”

## Supplementary Figures


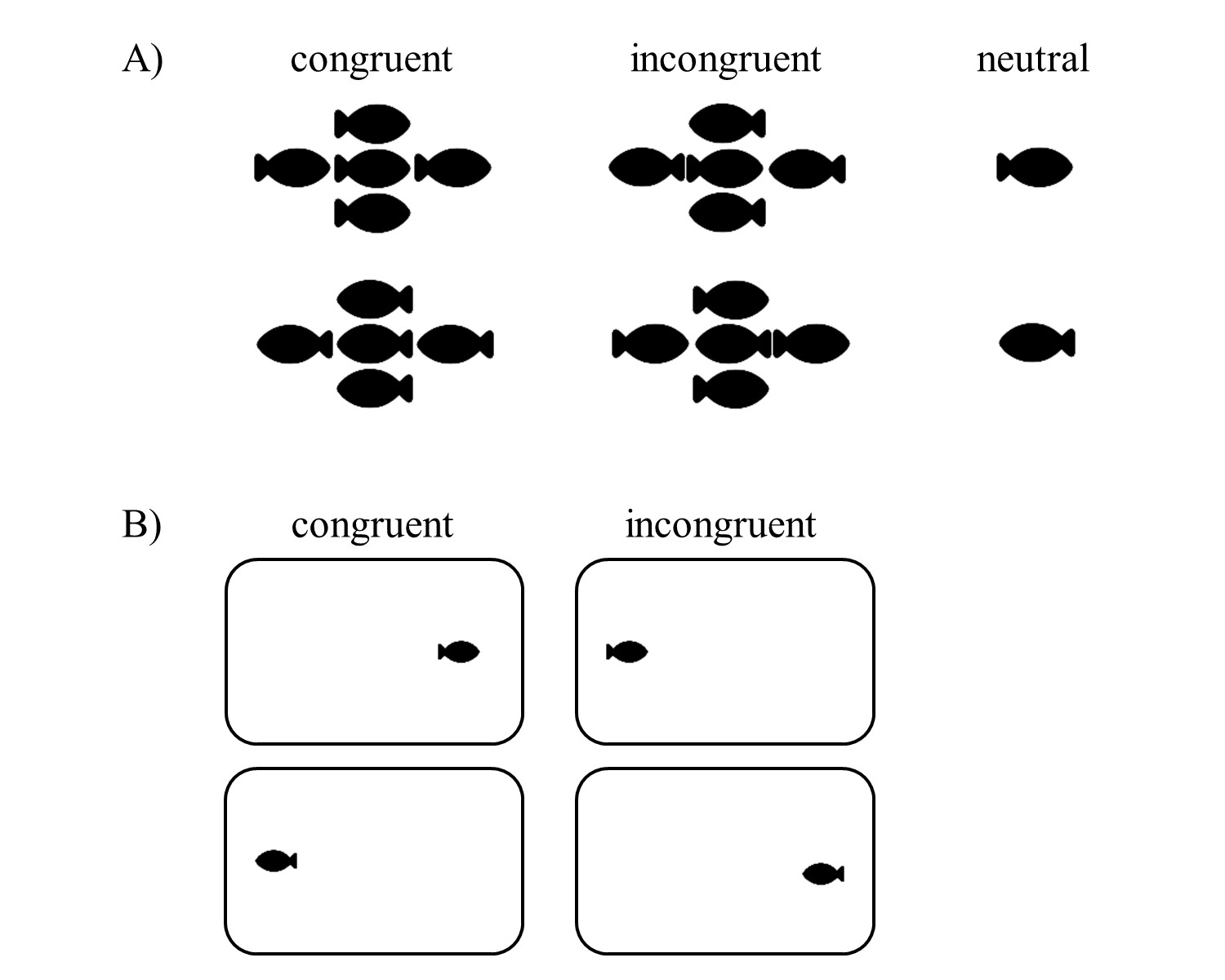


**Supplementary Figure 1.** (A) Examples of targets with congruent, incongruent, and neutral flankers. (B) Examples of trials with congruent and incongruent spatial stroop.


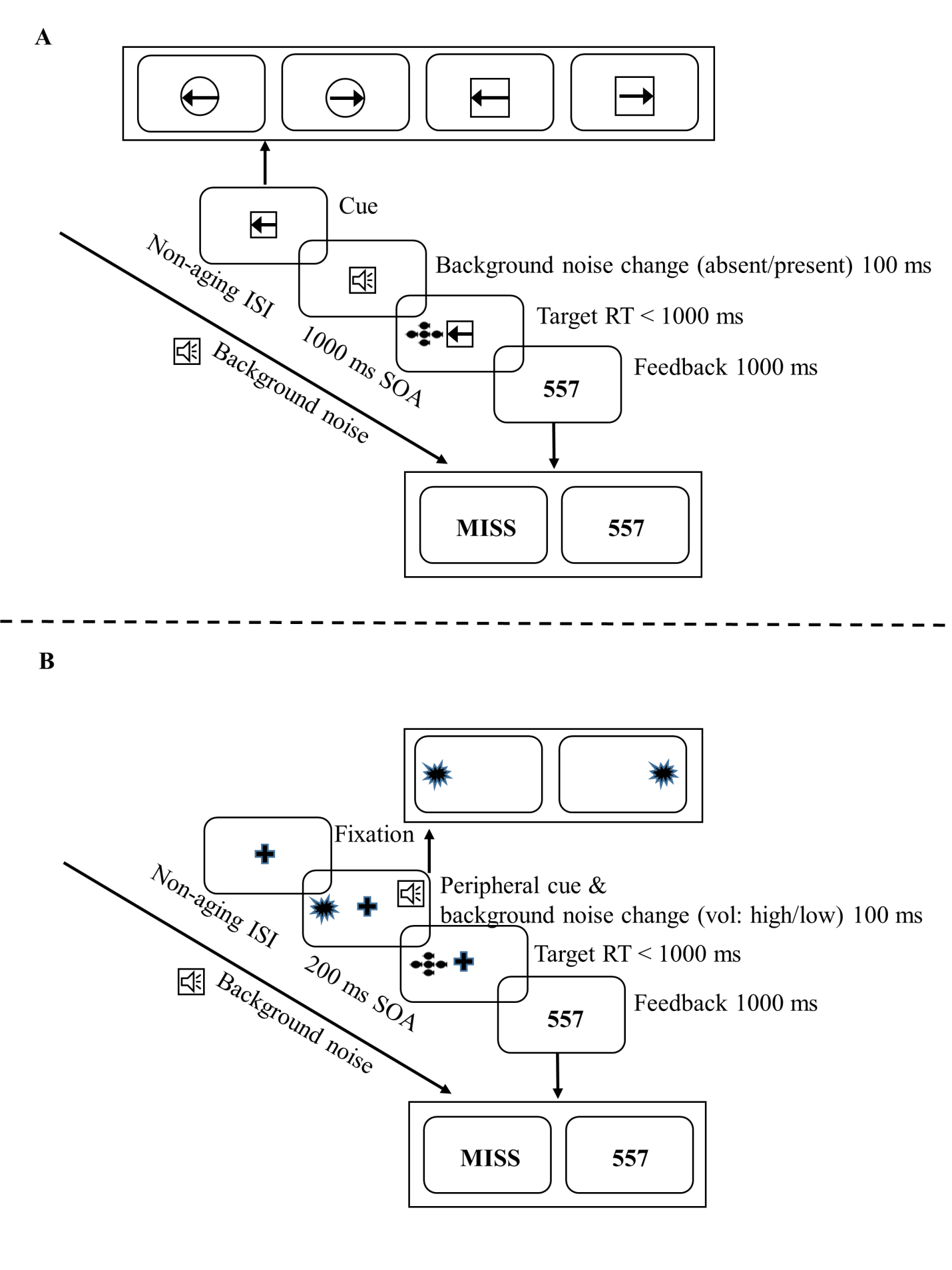


**Supplementary Figure 2.** A schematic representation of the Combined Attention Systems Test. (A) Experimental procedure of the endogenous task. (B) Experimental procedure of the exogenous task. ISI = inter stimulus interval; SOA = stimulus onset asynchrony; vol = volume.
